# Integrated Transcriptomic and Metabolomic Analysis Reveals the Metabolic Basis and Regulatory Networks of Triterpenoid Biosynthesis in *Ziziphus jujuba* Mill. cv. ‘Junzao’ Fruits at Different Harvest Times

**DOI:** 10.3390/foods15142427

**Published:** 2026-07-08

**Authors:** Yahui Yan, Wei Qin, Zuoshan Feng, Guoqiang Fu

**Affiliations:** 1 Key Laboratory of Postharvest Science and Technology for Fruit Products, College of Food Science and Pharmacology, Xinjiang Agricultural University, Urumqi 830052, China; fengzuoshan@126.com (Z.F.); 13569378263@163.com (G.F.); 2 Postdoctoral Station of Horticulture, Xinjiang Agricultural University, Urumqi 830052, China; xjqinwei@163.com; 3 College of Agriculture, Xinjiang Hetian College, Hetian 848000, China

**Keywords:** cytochrome P450, fruit development, gene co-expression network, metabolic regulation, WGCNA

## Abstract

Triterpenoids are the primary bioactive constituents responsible for the medicinal efficacy of *Ziziphus jujuba* Mill. cv. ‘Junzao’ (Junzao) fruits, and there are significant differences in their functional composition depending on the harvest period. However, the metabolic characteristics and dynamic accumulation patterns of triterpenoids during fruit development remain poorly understood. This study represented the first systematic integration of transcriptomic and metabolomic analysis combined with weighted gene co-expression network analysis (WGCNA) to elucidate the biosynthetic pathways of triterpenoids during the harvest times of Junzao fruits, and provide a scientific basis for the future development of functional foods. Total triterpenoid content exhibited a stage-specific accumulation pattern, peaking at the YG (young fruit) stage of fruit development, declining sharply by 37% at the PD (expansion) stage, rebounding at the BS (white-ripe) stage, and gradually decreasing through the CS (crispy-ripe) stage and WS (full-ripe) stages. A total of 347 terpenoid differentially accumulated metabolites (DAMs) and 18,925 differentially expressed genes (DEGs) were identified, among which 224 triterpenoids were predominant. A total of 347 terpenoid differentially accumulated metabolites (DAMs) and 18,925 differentially expressed genes (DEGs) were identified, among which 224 triterpenoids were predominant. WGCNA identified six key modules (salmon, midnightblue, black, blue, yellow, and brown modules) strongly correlated with the accumulation of methyl oleanolate, 3-oxopomolic acid, hederagenin, and other triterpenoids. Furthermore, integrated correlation analysis revealed that cytochrome P450 family genes, particularly CYP716, CYP72A and CYP88, were likely the hub genes governing triterpenoid biosynthesis and RT-qPCR validation of eight key genes confirmed the transcriptome expression trends. These findings provide a comprehensive framework for understanding triterpenoid biosynthesis and offer theoretical foundations for optimizing harvest timing and advancing metabolic engineering in Junzao fruits.

## 1. Introduction

*Ziziphus jujuba* Mill. is widely cultivated in China and represents an important medicinal-food homology resource in traditional Chinese medicine [1,2,3]. Jujuba fruits are rich in bioactive metabolites, including polysaccharides, flavonoids, terpenoids, and cyclic adenosine monophosphate, and are associated with diverse health-promoting properties such as antioxidant, immunomodulatory, hepatoprotective, and neuroprotective activities [4]. Jujubes have been widely cultivated in Xinjiang because this region offers the most pleasant climate and soil conditions for the plant [5]. *Ziziphus jujuba* Mill. cv. ‘Junzao’ (Junzao) is a primary jujube cultivar in Xinjiang, valued for its superior yield, outstanding stress tolerance, and favorable organoleptic properties [6]. Beyond its agronomic merits, this cultivar underpins a significant portion of the region’s dried-fruit and nutraceutical industry [7]. In plants, terpenoids perform essential physiological functions during plant growth, including serving as pollinator attractants, mediating defense responses against herbivores and pathogens, and acting as inter- and intra-plant signaling molecules [8,9].

Among the terpenoid constituents in jujube fruits, triterpenoids are considered a key chemical basis for the medicinal value of jujube fruits and an important criterion for quality evaluation [10]. Jujube triterpenoids mainly include oleanane-, ursane-, and lupane-type metabolites, such as oleanolic acid, ursolic acid, betulinic acid, and their glycosylated derivatives, including jujubosides A and B [11,12]. These metabolites exhibit a broad spectrum of bioactivities, and their pharmacological properties are closely related to structural diversity, encompassing anticancer, anti-inflammatory, hepatoprotective, anti-diabetes, and sedative effects [13,14,15,16,17]. Although triterpenoids have been identified in several jujube cultivars, most studies have focused on metabolite isolation, targeted quantification, or bioactivity evaluation. The metabolic characteristics of fruit secondary metabolites are often shaped by both cultivar background and growing environment. Unlike the cultivars examined in most previous studies, Xinjiang Junzao is cultivated under strong solar radiation, low precipitation, and large diurnal temperature variation, which may reshape the temporal dynamics and structural diversity of triterpenoid accumulation [18]. However, the dynamic triterpenoid metabolic profile of Xinjiang Junzao fruits during development and across different harvest times remains insufficiently characterized, and the metabolic features that distinguish Junzao from other jujube varieties are still largely unclear.

Triterpenoid biosynthesis proceeds mainly through the mevalonate (MVA) pathway, in which 3-hydroxy-3-methylglutaryl-CoA reductase (HMGR), farnesyl pyrophosphate synthase (FPS), squalene synthase (SQS), squalene epoxidase (SQE), and oxidosqualene cyclases (OSCs) catalyze the formation of squalene and 2,3-oxidosqualene, the common precursors of triterpenoid skeletons [19,20]. Further structural diversification is mediated mainly by cytochrome P450 monooxygenases (CYP450s) and glycosyltransferases [21]. CYP450 enzymes participate primarily in the post-modification of triterpenoids and play a key role in their structural diversity, particularly members of the CYP716, CYP72, and CYP88 families [22,23,24,25]. In jujube, several genes involved in triterpenoid biosynthesis have been reported, including ZjHMGR, ZjOSC, CYP93D1, UGT71A16, and several other CYP450 family members [21,23,26]. Nevertheless, the specific genes and regulatory networks responsible for triterpenoid accumulation in Junzao fruit remain largely unknown, particularly across different harvest times. Although triterpenoid-related metabolites and genes have been reported in jujube, whether Xinjiang Junzao exhibits distinct temporal accumulation patterns or unique biosynthetic regulation remains unresolved. Such information is necessary for understanding cultivar-specific quality formation and for improving the utilization of triterpenoid resources in jujube.

Integrated transcriptomic and metabolomic analysis has become a powerful approach for linking metabolic phenotypes with gene expression changes and identifying key regulatory genes in plant secondary metabolism [27]. In particular, weighted gene co-expression network analysis (WGCNA) enables the association of co-expressed gene modules with metabolite traits and has been widely used [28]. This strategy has been successfully applied in multiple plant species, including physalis [29], tea [30], and citrus [31], but has not yet been systematically used to dissect triterpenoid biosynthesis in Junzao fruit across harvest times.

Accordingly, this study addresses two questions for Xinjiang Junzao fruit: (i) How do triterpenoid metabolites change dynamically across fruit harvest times, and does it follow a cultivar-specific accumulation pattern? (ii) Which candidate genes and pathways are key regulators of triterpenoid biosynthesis? We hypothesized that triterpenoid accumulation in Xinjiang Junzao follows a stage-specific, non-linear pattern governed by the coordinated, developmentally regulated expression of terpenoid-backbone and CYP450-mediated modification genes. To test these hypotheses, we performed the first integrated transcriptomic and metabolomic analysis combined with WGCNA to investigate triterpenoid biosynthesis across five harvest times in Xinjiang Junzao. Specifically, we aimed to elucidate the dynamic changes in triterpenoid compounds and identify the cultivar-specific accumulation patterns they follow; determine the key genes and pathways involved in triterpenoid biosynthesis and construct a regulatory network associated with triterpenoid accumulation, with particular emphasis on CYP450 candidate genes. These findings provide new insights into the metabolic basis and regulatory mechanisms of triterpenoid biosynthesis in Xinjiang Junzao and offer a theoretical foundation for harvest optimization, quality evaluation, and future metabolic engineering of jujube resources.

## 2. Materials and Methods

### 2.1. Plant Materials and Sample Collection

**Figure 1:**
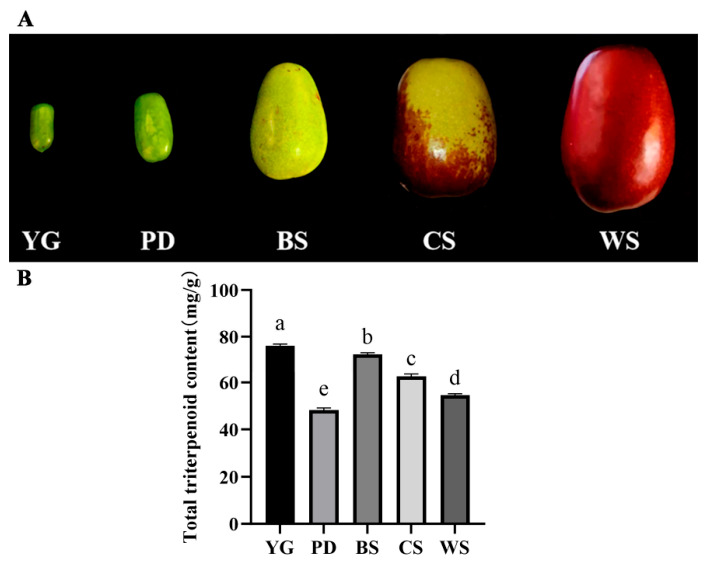
Morphological characteristics and total triterpenoid content of *Ziziphus jujuba* Mill. cv. ‘Junzao’ (Junzao) fruit at five harvest times: YG (young fruit) stage, PD (expansion) stage, BS (white-ripe) stage, CS (crispy-ripe) stage, and WS (full-ripe) stage. (**A**) Representative images of Junzao fruit. (**B**) Total triterpenoid content. Different letters above the bars indicate statistically significant differences (*p* < 0.05).

Junzao fruits were hand-harvested from the jujube germplasm resource nursery located in Maigaiti County, Kashgar, Xinjiang, China (N 38.99°, E 77.67°). Five harvest times were 28 June (YG, young fruit stage), 20 July (PD, expansion stage), 14 August (BS, white-ripe stage), 9 September (CS, crispy-ripe stage), and 2 October (WS, full-ripe stage), 2023 (Figure 1A). The samples were taken from 5-year-old Junzao trees grown under the same climatic conditions and management regime. A total of 30 jujube fruits were randomly harvested from one Junzao tree as one biological replicate, and three biological replicates were prepared for each stage. All materials were snap-frozen in liquid nitrogen in the field within 10 min after hand-harvesting, transported to the laboratory on dry ice (transit time 15 h), and stored at −80 °C until extraction. All experiments in this study were conducted using three replicates.

### 2.2. Determination of Total Triterpenoid Content

The total triterpenoid content was quantified via a modified method based on previous studies [32,33]. Briefly, 1.0 g of accurately weighed freeze-dried sample powder was extracted with 40 mL of 90% ethanol (*v*/*v*) via ultrasonic-assisted extraction for 45 min at room temperature, and centrifuged at 8000 r/min for 15 min. The extraction procedure was repeated twice, and the supernatants were combined for further analysis. For colorimetric analysis, 1 mL aliquots of the supernatant or standard oleanolic acid standard solutions (0–0.12 mg) were evaporated to dryness in a 90 °C water bath. The residues were then reacted with 0.2 mL of freshly prepared 5% (*w*/*v*) vanillin-glacial acetic acid solution and 0.8 mL of perchloric acid at 60 °C for 20 min. Further, after immediate cooling, the reaction mixture was diluted with 5 mL of glacial acetic acid, and the absorbance was measured at 548 nm using a UV-Vis spectrophotometer (Beijing Puxi General Instrument Co., Ltd., Beijing, China). The standard calibration curve (y = 0.0097x − 0.0016) was established using oleanolic acid, yielding an R^2^ of 0.9994 (Figure S1), and all measurements were performed in triplicate.

### 2.3. Metabolite Analysis

#### 2.3.1. Metabolite Extraction

The fresh Junzao fruits were subjected to vacuum freeze-drying using a ScientZ-100F lyophilizer (Ningbo Scientz Biotechnology Co., Ltd., Ningbo, China), and homogenized into fine powder via a Retsch MM 400 ball mill (Retsch GmbH, Haan-Gruiten, Germany) at 30 Hz for 1.5 min. Precisely weighed portions of the powder (50 mg) were mixed with 2.5 mL of precooled (−20 °C) 70% methanol through six cycles of vortex mixing (30 s at 3000 rpm) at 30 min intervals at 4 °C, then centrifuged at 12,000× *g* for 3 min (4 °C). The supernatant was filtered through a 0.22 μm PTFE membrane (Merck Millipore, Burlington, MA, USA) and stored at 4 °C until analysis.

#### 2.3.2. UPLC-MS/MS Analysis

Metabolite analysis was performed following the method described by Sun et al. [34]. Chromatographic separation was conducted on an ExionLC™ AD UPLC system (SCIEX, Fremont, CA, USA) coupled with an Agilent SB-C_18_ column (1.8 μm particle size, 2.1 × 100 mm) at 40 °C. The mobile phase consisted of (A) 0.1% formic acid in ultrapure water and (B) 0.1% formic acid in acetonitrile at 0.35 mL/min. The gradient elution was set as follows: 0–9 min, 5–95% B; 9–10 min; 95% B; 10–11 min, 95–5%B; 12–14 min, 5%B. The injection volume was 2 μL. Mass spectrometry was performed on an Applied Biosystems 4500 QTRAP system (SCIEX, USA) with an ESI Turbo Ion-Spray interface in both positive and negative ionization modes, controlled by Analyst 1.6.3 software. ESI source parameters: ionization temperature 550 °C; ion spray voltages +5500 V (positive) and −4500 V (negative); gas flows 50 psi (GSI), 60 psi (GSII), and 25 psi (curtain gas).

#### 2.3.3. Metabolomic Data Processing and Analysis

Metabolite identification was performed against the Metware in-house standard database (MWDB), which is constructed from authentic reference standards. Identification was based on the combination of accurate precursor (Q1) and product (Q3) ion pairs in MRM mode, secondary (MS/MS) fragmentation spectra, and retention-time matching, with MS and MS2 tolerances of 20 ppm and an RT tolerance of 0.2 min. Metabolites matched to authentic standards in the MWDB were assigned Level 1 (mass score > 0.7) > Level 2 (mass score = 0.5~0.7), and Level 3 (mass score < 0.5), with accuracy decreasing in that order. Differentially accumulated metabolites (DAMs) of PD vs. YG, BS vs. PD, CS vs. BS, and WS vs. CS were screened based on VIP ≥ 1 and |Fold change| ≥ 2 or |Fold change| ≤ 0.5 [35,36]. Peak areas of identified metabolites were used for principal component analysis (PCA), Pearson correlation coefficients (PCC), K-means clustering, hierarchical clustering analysis (HCA), and orthogonal partial least squares discriminant analysis (OPLS-DA) using R packages 1.0.1. To avoid overfitting, 200 permutations were performed. Venn diagram construction and Kyoto Encyclopedia of Genes and Genomes (KEGG) pathway enrichment analysis were performed using Metware Cloud (https://cloud.metware.cn (accessed on 12 November 2025)).

### 2.4. Transcriptomics Analysis

Transcriptome sequencing was performed by Wuhan Metware Biotechnology Co., Ltd. (Wuhan, China). Total RNA was extracted from frozen fruit samples using a Tissue RNA Extraction Kit 2.0 Plus (Vazyme Biotech Co., Ltd., Nanjing, China) according to the manufacturer’s instructions. Transcriptome libraries were constructed using the NEBNext Ultra RNA Library Prep Kit and sequenced on the MGI platform (Metware Biotechnology). After removing adapters and low-quality reads, clean reads were aligned to the jujube reference genome (genome00479) using HISAT2 (v2.2.1) [37]. Gene expression levels were represented as FPKM values. Differential expression analysis was performed using DESeq2 and edgeR. Genes with FDR < 0.05, |log2 fold change| ≥ 1, and Padj < 0.05 were considered differentially expressed genes (DEGs) [36].

### 2.5. Integrated Analysis of Metabolome and Transcriptome

Gene expression data (FPKM values) and triterpenoid metabolite abundances were used as input for co-expression network construction using the WGCNA package in 1.71. The soft-thresholding power was determined to ensure scale-free network topology. Modules were identified using the dynamic tree cut method (MergeCutHeight = 0.25, MinModuleSize = 50). Module eigengenes (MEs) were calculated as the first principal component of each module, and their correlations with triterpenoid contents were assessed by Pearson correlation analysis. The selection of the key module was based on the highest correlation with triterpenoid content and a biologically meaningful expression pattern. Modules with |r| > 0.7 and *p* < 0.05 were selected for further KEGG pathway enrichment analysis.

### 2.6. Quantitative Real-Time PCR (qRT-PCR) Validation

Eight key genes involved in triterpenoid biosynthesis were selected for qRT-PCR validation. Gene-specific primers were designed using NCBI Primer-BLAST tool (http://www.ncbi.nlm.nih.gov/tools/primer-blast/ (accessed on 23 June 2026)) and synthesized by Tsingke Biotech Co., Ltd. (Beijing, China). Total RNA was reverse-transcribed using the PrimeScript RT Reagent Kit with gDNA Eraser (Takara Bio Inc., Kusatsu City, Shiga Prefectur, Japan). The qRT-PCR was performed on a Bio-Rad CFX96 Touch™ Real-Time PCR Detection System using Takara TB Green^®^ Premix Ex Taq™ II (Tli RNaseH Plus) (Takara Bio Inc., Kusatsu City, Shiga Prefectur, Japan), according to the manufacturer’s protocol. The thermal cycling conditions were as follows: 95 °C for 30 s, followed by 40 cycles of 95 °C for 10 s and 60 °C for 30 s. The relative expression levels were calculated using the 2^−ΔΔCt^ method with ZjUBQ1 as the reference gene [38]. All reactions were performed in three biological and three technical replicates. The specific primer sequences are listed in Table S1.

### 2.7. Statistical Analysis

One-way ANOVA (*p* < 0.05) was performed using SPSS 26.0 (IBM Corp.Armonk, NY, USA). Data are presented as mean ± SD of three biological replicates. Figures were generated using GraphPad Prism 13.0 (GraphPad Software, San Diego, CA, USA).

## 3. Results

### 3.1. Differences in the Total Triterpenoid Content Across Junzao Fruits with Different Harvest Times

To investigate the dynamic changes in triterpenoid accumulation during jujube fruit development, we collected Junzao fruits at five distinct harvest times (YG, PD, BS, CS, WS) (Figure 1A). The total triterpenoid content exhibited a significant dynamic pattern during fruit development (Figure 1B). The highest triterpenoid content was observed at the YG stage (76.48 ± 0.51 mg/g), followed by a sharp decrease to (48.04 ± 0.79) mg/g at the PD stage, representing a 37% reduction. Subsequently, the triterpenoid content increased to (72.19 ± 0.53) mg/g at the BS stage, then gradually decreased to (62.17 ± 0.39) mg/g at the CS stage and (54.55 ± 0.65) mg/g at the WS stage. Statistical analysis indicated significant differences across harvest times (*p* < 0.05), suggesting that triterpenoid biosynthesis and accumulation follow a stage-specific pattern, with the most dramatic change occurring during the YG-to-PD transition.

### 3.2. Metabolomic Profiling Reveals Dynamic Changes in Terpenoid Metabolites

**Figure 2:**
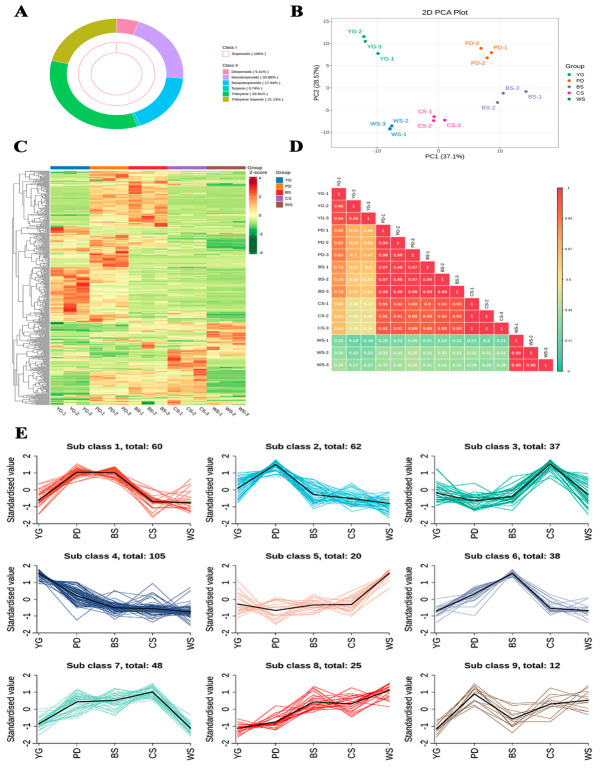
Multivariate statistical analysis of metabolites in Junzao fruits at five development times. (**A**) Distribution of metabolites. (**B**) Principal Component Analysis (PCA) of metabolomic data. (**C**) Hierarchical clustering analysis (HCA) of secondary metabolites. (**D**) Correlation analysis. (**E**) K-means clustering of the metabolites.

To comprehensively understand the terpenoid metabolic landscape of Junzao fruits, we performed global metabolomic analysis across the five harvest times by UPLC-MS/MS. Total ion current (TIC) diagrams and multi-peak detection plots of one quality control (QC) sample are shown in Figure S2. A total of 407 terpenoid metabolites were identified across 6 major groups (Figure 2A, Table S2), including 224 triterpenoids (138 triterpene (33.91%) and 86 triterpene saponin (21.13%)), monoterpenoids (85, 20.88%), sesquiterpenoids (73, 17.94%), ditepenoids (22, 5.41%), and terpene (3, 0.73%). PCA revealed that significant differences between harvest times, with no significant differences within the group, confirming data reliability (Figure 2B). PC1 and PC2 explained 64.8% of the total variance (37.1% and 27.7%, respectively), with YG and PD stages clustering separately from later stages (BS, CS, WS), indicating distinct metabolic profiles between early and late harvest times. HCA further illustrated temporal metabolic variations, with PD and CS samples showing substantially higher terpenoid metabolite abundance (Figure 2C). Pearson correlation analysis confirmed strong positive correlations within the same harvest times and weaker correlations between different times (Figure 2D). Additionally, metabolites were further clustered into nine subclasses by K-means clustering (Figure 2E, Table S3). Triterpenoids were primarily concentrated in subclass 1, subclass 4 and subclass 7. Collectively, HCA and K-means analyses delineated fruit development into three main metabolic phases: early (YG), middle (PD, BS), and late (CS, WS). Subsequently, OPLS-DA models clearly separated samples from different stages, consistent with PCA and HCA results (Figure S3A–D). All models showed Q^2^ > 0.95, confirming model reliability (Figure S3E–H).

**Figure 3:**
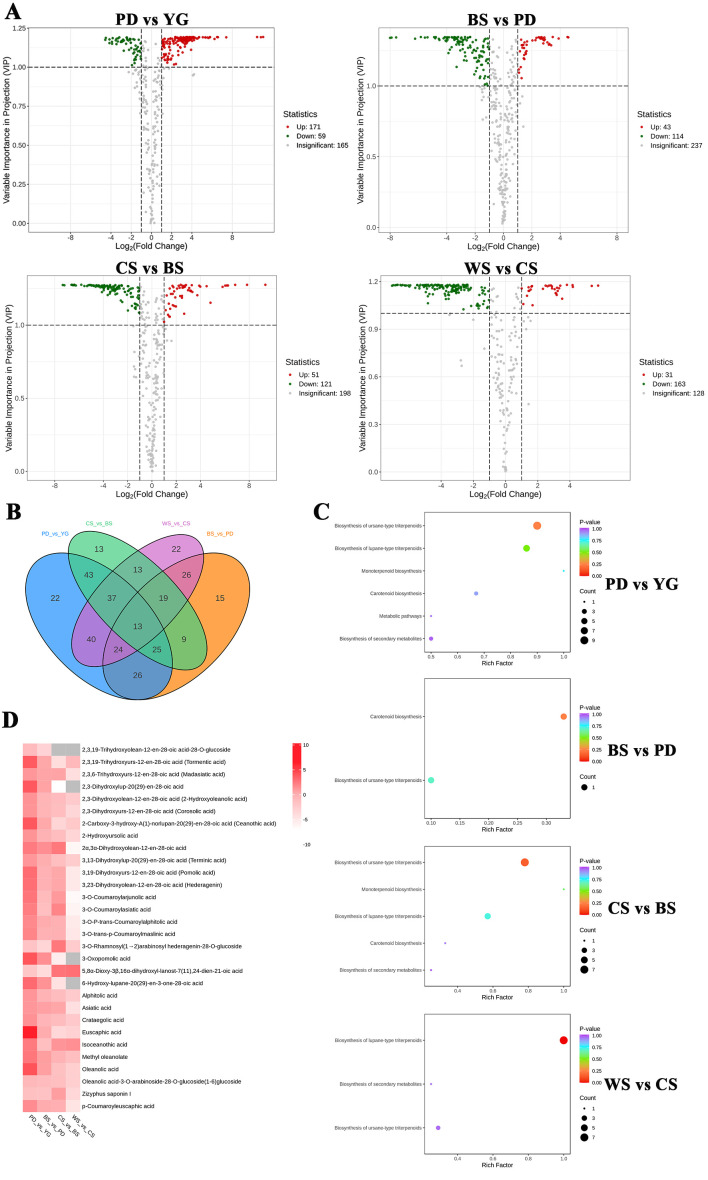
Differentially accumulated metabolites (DAMs) analysis of Junzao fruits at five development times: (**A**) Volcano plots showing DAMs in pairwise comparisons. (**B**) Venn diagram of DAMs among four pairwise comparisons. (**C**) Heatmap showing the relative abundance of the key metabolites involved in the triterpenoid biosynthesis pathway across five harvest times. (**D**) Kyoto Encyclopedia of Genes and Genomes (KEGG) analysis of multiple combinations of DAMs in pairwise comparisons.

A total of 347 DAMs were detected through pairwise comparisons of adjacent harvest times, with upregulated and downregulated metabolite numbers in each comparison summarized in Figure 3A and Table S4. The highest number of DAMs was observed in PD vs. YG (230 DAMs), while the majority of DAMs showed downregulation during later harvest times. Venn diagram analysis revealed a core set of 13 common terpenoid DAMs across all four comparisons, of which 11 were triterpenoid metabolites, including 3-O-coumaroylasiatic acid, isoceanothic acid, 2α,3α-dihydroxyolean-12-en-28-oic acid, and 3-O-rhamnosyl (1 → 2) arabinosyl hederagenin-28-O-glucoside, and other triterpenoids. (Figure 3B, Table S5), suggesting stage-specific metabolic shifts at each developmental transition.

KEGG pathway enrichment analysis of DAMs revealed significant enrichment in triterpenoid biosynthesis-related pathways, including ursane-type triterpenoid biosynthesis (MetMap175), lupane-type triterpenoid biosynthesis (MetMap176) (Figure 3C). Ursane-type triterpenoid biosynthesis was the top-enriched pathway in PD vs. YG and CS vs. BS comparisons, while lupane-type triterpenoid biosynthesis was predominant in WS vs. CS, reflecting stage-specific shifts in triterpenoid metabolism. Further screening identified 30 key DAMs related to triterpenoid biosynthesis, including oleanolic acid, asiatic acid, alphitotic acid, and euscaphic acid and their derivatives, showing stage-specific accumulation patterns (Figure 3D).

### 3.3. Transcriptomic Analysis Reveals Gene Expression Patterns Associated with Fruit Development

**Figure 4:**
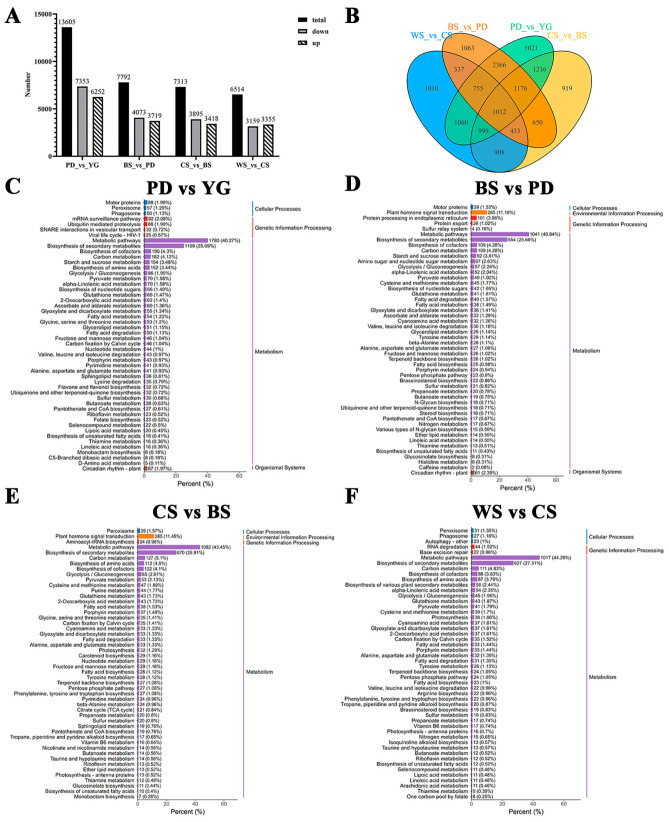
Transcriptomic analysis of Junzao fruits at five development stages: (**A**) Bar plot showing the number of DEGs identified in pairwise comparisons. (**B**) Venn diagram of pairwise comparison of DEGs. (**C**–**F**) KEGG pathway enrichment analysis of DEGs in pairwise comparisons.

RNA-seq analysis was performed across five harvest times to elucidate the molecular mechanisms underlying triterpenoid accumulation in Junzao fruits. Sequencing quality control analysis showed Q20 and Q30 base percentages of 99–99.31% and 96.54–97.41%, respectively, with GC content ranging from 42.68% to 44.46%, confirming high data quality (Table S6). A total of 18,925 DEGs were identified across four pairwise comparisons: 6252 up-regulated and 7353 down-regulated in PD vs. YG; 3719 up-regulated and 4073 down-regulated in BS vs. PD; 3418 up-regulated and 3895 down-regulated in CS vs. BS; and 3355 up-regulated and 3159 down-regulated in WS vs. CS (Figure 4A, Table S7). The highest number of DEGs was observed in PD vs. YG, with numbers declining progressively during later ripening stages, consistent with metabolomic changes. Among all comparisons, 1012 common DEGs were shared, while 5021, 1063, 919, and 1010 DEGs were unique to each respective comparison (Figure 4B).

**Figure 5:**
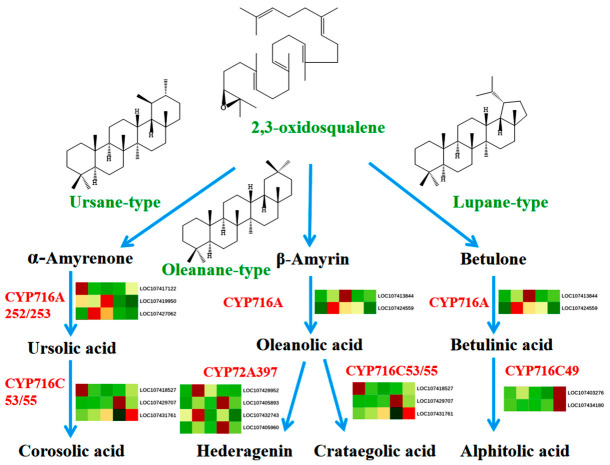
Schematic representation of the main triterpenoid biosynthesis pathways in Junzao fruits, integrated with gene expression patterns. The diagram illustrates the conversion of 2,3-oxidosqualene into different triterpenoid skeletons (Ursane-type, Oleanane-type, and Lupane-type) and their subsequent downstream metabolites.

KEGG pathway enrichment analysis revealed significant enrichment in triterpenoid-related pathways throughout fruit development, including ubiquinone and other terpenoid-quinone biosynthesis (ko00130), terpenoid backbone biosynthesis (ko00900), sesquiterpenoid and triterpenoid biosynthesis (ko00909), diterpenoid biosynthesis (ko00904), and monoterpenoid biosynthesis (ko00902) (Figure 4C–F). ko00900 supplies the universal C5 precursors for all terpenoids, while ko00909 directly encompasses triterpenoid skeleton formation and modification [39,40]. Overall, triterpenoid regulation does not occur in isolation but is part of a coordinated modulation of the terpenoid metabolic network. Six DEGs involved in the triterpenoid biosynthesis pathway were identified: CYP72A397 (LOC107405893, LOC107405960, and LOC107432743), CYP716A (LOC107413844), CYP716C49 (LOC107403276), and CYP716C53/55 (LOC107418527). Integrated visualization of gene expression and metabolite accumulation across harvest times demonstrated that CYP450 family members exhibited dynamic expression patterns closely mirroring the accumulation trends of specific triterpenoids (Figure 5). Pathway analysis confirmed that 2,3-oxidosqualene serves as a critical branch point for ursane-type, oleanane-type, and lupane-type triterpenoid biosynthesis [41,42], with CYP716 and CYP72 family members acting as key regulators throughout Junzao fruit development.

### 3.4. WGCNAAnalysis 

**Figure 6:**
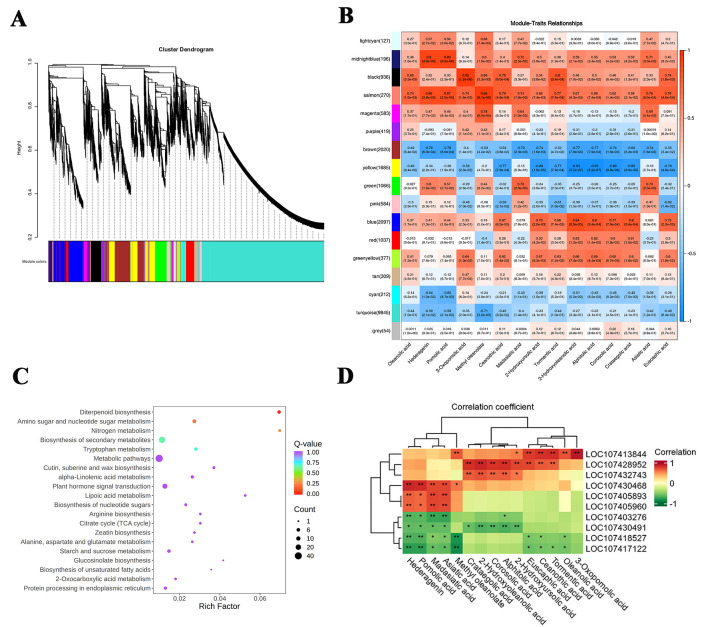
Co-expression network analysis in triterpenoid biosynthesis. (**A**) Clustering dendrogram of DEGs, with dissimilarity based on the topological overlap, together with assigned module colors. (**B**) Module-trait associations. (**C**) KEGG pathway enrichment analysis of DEGs in the brown module. (**D**) Correlation heatmap showing the relationships between the expression levels of key triterpenoid biosynthesis genes and the contents of specific triterpenoids. * Indicates correlations between the variables (* *p* < 0.05, and ** *p* < 0.01).

To unravel the regulatory mechanisms governing triterpenoid biosynthesis during Junzao fruit development, transcriptomic and metabolomic datasets were integrated through WGCNA to identify key gene-metabolite associations. The clustering dendrogram revealed distinct co-expressed gene modules (Figure 6A). DEGs were partitioned into seventeen modules with distinct expression patterns (Table S8). Module-trait correlation analysis identified six modules showing the highest correlations with fifteen major triterpenoid DAMs: salmon, midnightblue, black, and blue modules exhibited the highest positive correlation coefficients, while yellow and brown modules displayed negative correlations with major triterpenoids (Figure 6B). Six module genes have their own specific expression patterns (Figure S4).

KEGG enrichment analysis of these six modules revealed significant enrichment in triterpenoid biosynthetic pathways, including diterpenoid biosynthesis, sesquiterpenoid and triterpenoid biosynthesis, and terpenoid backbone biosynthesis, with the brown module significantly enriched in diterpenoid biosynthesis, consistent with DEGs enrichment results. (Figure 6C and Figure S5). Through pathway analysis, CYP88A (LOC107430468) and CYP88D6 (LOC107430491) were identified as additional key regulatory factors. Gene-metabolite correlation analysis between fifteen candidate genes and fifteen triterpenoid metabolites confirmed strong correlations (Figure 6D, Table S9). Collectively, three CYP72 genes, three CYP716 genes, and two CYP88 genes were identified as likely participants in Junzao triterpenoid biosynthesis and regulation.

### 3.5. Validation of Key Gene Expression Patterns

**Figure 7:**
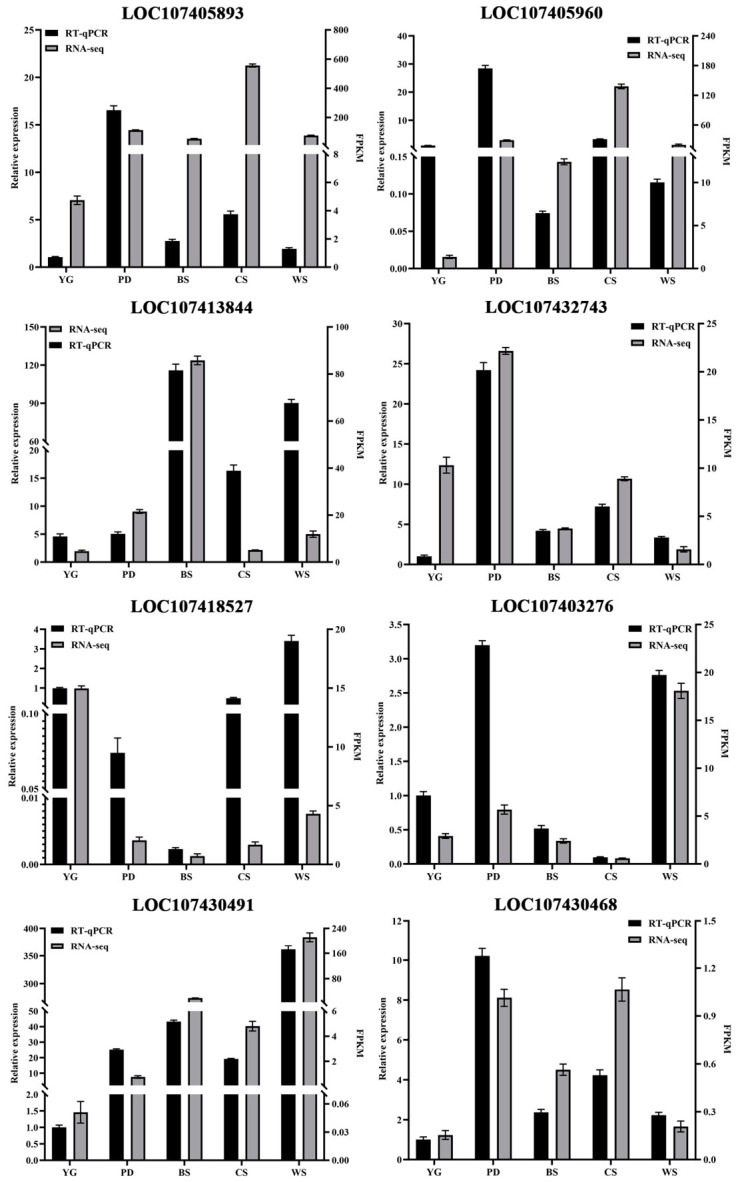
Validation of RNA-seq data by qRT-PCR analysis of eight selected DEGs involved in triterpenoid biosynthesis.

To validate the RNA-seq data, eight selected DEGs were subjected to qRT-PCR across the five harvest times (Figure 7). The expression profiles obtained by qRT-PCR showed strong consistency with RNA-seq data for all selected genes, with most genes displaying the highest expression during early harvest times (YG), corresponding to the period of active triterpenoid biosynthesis, thus confirming the reliability of the identified DEGs and their roles in the developmental regulation of triterpenoid biosynthesis in Junzao fruits.

## 4. Discussion

The postharvest quality of jujube fruits is intrinsically linked to their bioactive constituents, with triterpenoids representing the primary material basis for medicinal efficacy and serving as critical quality evaluation indices [43]. This study integrated metabolomics and transcriptomics to systematically elucidate the dynamic accumulation patterns of triterpenoids and their underlying molecular regulatory mechanisms in *Z. jujuba* cv. Junzao fruits across five harvest times, revealing a distinctive “dual-peak” accumulation pattern, key genes governing triterpenoid skeletal diversification, and gene co-expression networks associated with triterpenoid metabolism.

In the present study, the total triterpenoid content in Junzao fruits exhibited a pronounced developmental dynamic, reaching its highest level at the YG stage, then declining sharply by 37% at the PD stage, rebounding at the BS stage, and subsequently decreasing through the CS and WS stages. The accumulation pattern featured a first peak at the YG stage, consistent with a chemical-defense role in which high triterpenoid levels provide antimicrobial and anti-herbivore protection for vulnerable immature tissues [19]; a pronounced PD decline during fruit enlargement, likely attributable to a dilution effect and a redirection of carbon/metabolic flux toward rapid cell expansion and primary metabolism; and a second peak at the BS transition, which coincided with the onset of ripening-associated secondary metabolism and identified BS as a critical transition point for triterpenoid accumulation [44]. The “dual-peak” accumulation pattern observed in Xinjiang Junzao cultivars provides novel insights into triterpenoid metabolism. This pattern further delineates two harvest windows—YG and BS—that maximize the accumulation of medicinally or nutraceutically relevant triterpenoids, thereby providing a scientific basis for stage-specific harvesting decisions and graded processing strategies. The high triterpenoid accumulation observed during early harvest times aligns with findings from other jujube cultivars: Pan et al. [45] also demonstrated significant fluctuations in triterpenoid content around the white-mature stage across multiple jujube cultivars, suggesting non-linear accumulation patterns may represent a conserved feature across ziziphus species; Shen et al. [46] similarly found sharply increased triterpenoid levels between the YF and WM stages of “Jinsixiaozao”. The differences in triterpenoid bioactive metabolites in jujube are largely influenced by cultivar, geographical environment, processing, and storage conditions [47].

The comprehensive metabolomic profiling in this study identified 224 triterpenoids, demonstrating the remarkable chemical diversity of the triterpenoid metabolic network in Junzao fruits. This extensive coverage exceeds previously reported triterpenoid inventories in jujube. Pan et al. [45] identified 85 triterpenoid metabolites across different jujube cultivars, while Zheng et al. [48] identified 74 dammarane-type and oleanane-type triterpenoids in *Ziziphus jujuba* var. spinosa. The expanded terpenoid metabolite discovered in our study included both active ingredients reported in the literature and new candidate functional molecules, which have potential guiding significance for food functional improvement. Meanwhile, the cross-omics integration strategy demonstrated the advantages of exploring complex biological systems. The three major skeletal types of triterpenoid metabolites identified (oleanane-type, ursane-type, and lupane-type triterpenoids) exhibited distinct and complementary pharmacological activities, including anti-inflammatory, antidiabetic, and antitumor properties that are directly relevant to jujube’s medicinal applications [49,50,51,52,53].

Some progress has been made in the functional validation of triterpenoid biosynthesis genes in jujube. Wen et al. [21] identified key candidate genes, including ZjFPS, ZjSQS1, ZjOSC1, ZjAACT1, and CYP450/3, contributing to triterpenoid synthesis in wild and cultivated jujube. Bai et al. [23] revealed that CYP93D1 and UGT71A16 are potential key genes in jujuboside B1 biosynthesis. Among biosynthetic enzymes, Cytochrome P450 monooxygenases play central roles in the downstream oxidative modification of triterpenoid scaffolds, mediating structural diversity that underpins diverse pharmacological activities [54]. CYP716A subfamily members are particularly recognized for adding a carboxyl group at the C-28 position of β-amyrin, leading to the production of oleanolic acid [55]. Sandeep et al. [56] reported that CYP716C55 catalyzed the C-2α hydroxylation of ursolic acid and oleanolic acid to produce corosolic acid and maslinic acid, respectively. Our results showed that CYP716A (LOC107413844), CYP716C49 (LOC107403276), and CYP716C53/55 (LOC107418527) expression patterns positively correlated with the accumulation of hydroxylated triterpenoids, including crataegolic acid and corosolic acid, suggesting these genes perform analogous catalytic functions in Junzao. The CYP72A subfamily contributes to additional skeletal modifications in triterpenoid saponin biosynthesis [57]. Biazzi et al. [58] identified CYP72A67 in *Medicago truncatula* catalyzing C-23 hydroxylation of oleanolic acid to generate hederagenin, while Han et al. [59] further characterized CYP72A397 as an oleanolic acid 23-hydroxylase involved in hederagenin production in *Kalopanax septemlobus*. Su et al. [60] reported that MaCYP88A108 and MaCYP88A164 function as β-amyrin 11-oxidases with dual functionality in pentacyclic triterpene and limonoid biosynthesis. Additionally, Bakhtiar et al. [61] revealed the strong positive correlations between CYP88D6 and the accumulation of glycyrrhizic acid, which reflected a conserved regulatory pattern in *Glycyrrhiza glabra* L. In our study, three CYP72A397 paralogs (LOC107405893, LOC107432743, and LOC107405960), CYP716A (LOC107413844), CYP716C49 (LOC107403276), CYP716C53/55 (LOC107418527), CYP88A (LOC107430468), and CYP88D (LOC107430491) were identified, expanding the known phytochemical diversity of this species and providing candidate targets for future functional validation and metabolic engineering applications.

Weighted gene co-expression network analysis has emerged as a powerful tool for identifying gene modules and hub genes associated with specific metabolite accumulation patterns in medicinal plants [62,63]. Meng et al. [64] have established yeast- and tobacco-based platforms for functional verification of triterpenoid CYP450 genes, which can be directly applied to validate the CYP716 and CYP72A genes identified herein. In the present study, WGCNA identified four positively correlated modules and two negatively correlated modules as being highly correlated with triterpenoid accumulation. This bidirectional regulatory pattern reflects the dynamic balance between biosynthesis and catabolism or metabolic flux redistribution during fruit development.

Despite the progress achieved, several limitations should be acknowledged. First, the catalytic functions of the candidate CYP450 genes require validation through in vitro enzyme assays or heterologous expression systems, and the roles of transcription factors in regulating triterpenoid biosynthesis networks warrant deeper investigation. Second, environmental factors influencing triterpenoid accumulation were not examined in this study, and optimization of genetic transformation systems for Junzao remains necessary for functional gene validation. Future research should focus on functional verification of the identified CYP72A397, CYP716, and CYP88 family candidate genes, elucidation of transcription factor-target gene regulatory relationships, and investigation of genotype-environment interactions affecting triterpenoid metabolism.

## 5. Conclusions

In conclusion, this study systematically elucidated the metabolic basis and regulatory networks of triterpenoid biosynthesis in Junzao fruits during different harvest times through integrated metabolomic and transcriptomic approaches. The distinctive “dual-peak” accumulation pattern, with maximum triterpenoid content at the YG stage, provides a theoretical foundation for quality-oriented harvest timing. The identification of CYP716A, CYP716C49, CYP716C53/55, CYP72A397, CYP88A, and CYP88D as hub genes governing triterpenoid skeletal diversification, together with six WGCNA modules associated with triterpenoids, establishes candidate targets for future metabolic engineering applications in Junzao fruits. These findings advance our understanding of triterpenoid biosynthesis in jujube and provide molecular targets for the metabolic engineering of triterpenoid production in Xinjiang jujube fruits.

## Data Availability

The original contributions presented in this study are included in the article/Supplementary Materials. Further inquiries can be directed to the corresponding authors.

## Supplementary Materials

The following supporting information can be downloaded at: https://www.mdpi.com/article/10.3390/foods15142427/s1, Figure S1: Standard curve for total triterpenoid content; Figure S2: The total ion current (TIC) chromatograms for the quality control (QC) sample (Positive ion mode and Negative ion mode); Figure S3: (A–D) Orthogonal partial least squares discriminant analysis (OPLS-DA) scores plots showing differentially accumulated metabolites (DAMs) in pairwise comparisons: (A) PD vs. YG, (B) BS vs. PD, (C) CS vs. BS, (D) WS vs. CS. (E-H) OPLS-DA model permutation in pairwise comparisons: (E) PD vs. YG, (F) BS vs. PD, (G) CS vs. BS, (H) WS vs. CS; Figure S4: Gene expression patterns of salmon, midnightblue, black, blue, brown, and yellow modules by weighted gene co-expression network analysis (WGCNA), respectively; Figure S5: KEGG analysis of midnightblue, blue, yellow, salmon, and black modules by WGCNA, respectively. The size of dots represents the number of metabolites enriched in each pathway, and the color indicates the significance level; Table S1: The specific primer sequences; Table S2: All terpenoid metabolites detected in *Ziziphus jujuba* Mill. cv. ‘Junzao’ (Junzao) fruits during different harvest times; Table S3: Obtain the distribution of terpenoid metabolites in each sub cluster through K-Means analysis; Table S4: Volcano plots showing differentially accumulated metabolites (DAMs) in pairwise comparisons; Table S5: Common DAMs of venn diagram among four pairwise comparisons (PD vs. YG, BS vs. PD, CS vs. BS, and WS vs. CS); Table S6: Quality assessment data for transcriptome sequencing; Table S7: The data results of differentially expressed genes (DEGs) by transcriptome analysis; Table S8: Distribution information of network nodes in each module by weighted gene co-expression network analysis (WGCNA); Table S9: Gene-metabolite correlation analysis between candidate genes and key triterpenoid metabolites.
