# Supplementary material for: Integrated Transcriptomic and Metabolomic Analysis Reveals the Metabolic Basis and Regulatory Networks of Triterpenoid Biosynthesis in Ziziphus jujuba Mill. cv. ‘Junzao’ Fruits at Different Harvest Times
Source: Foods. 2026 Jul 8;15(14):2427. doi: 10.3390/foods15142427 (PMC13409704; doi:10.3390/foods15142427)
Supplement: Supplementary file 1 [file foods-15-02427-s001.zip › Supplementary Figures S1-S5.pdf]

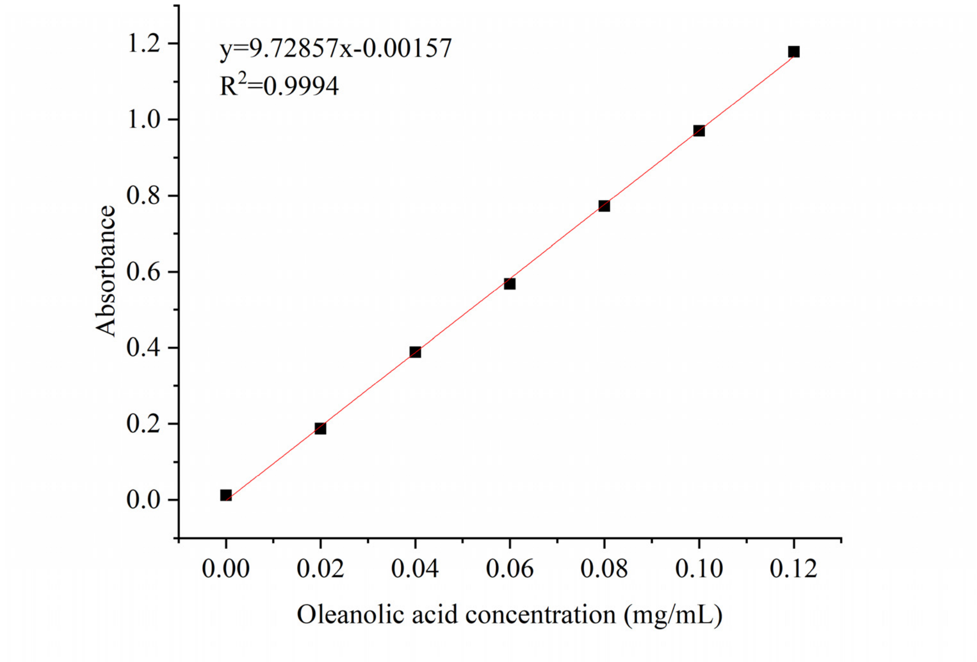

Figure S1. Standard curve for total triterpenoid content.

Postive iron

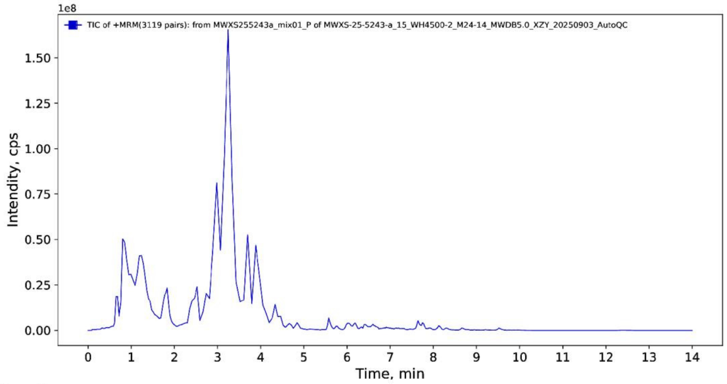

Negative iron

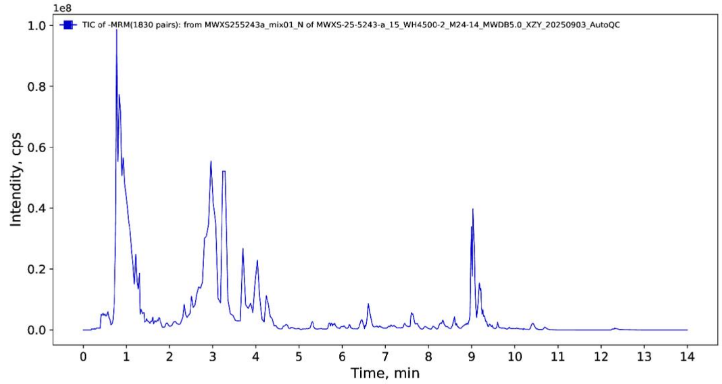

Figure S2. The total ion current (TIC) chromatograms for the quality control (QC) sample (Positive ion mode and Negative ion mode).

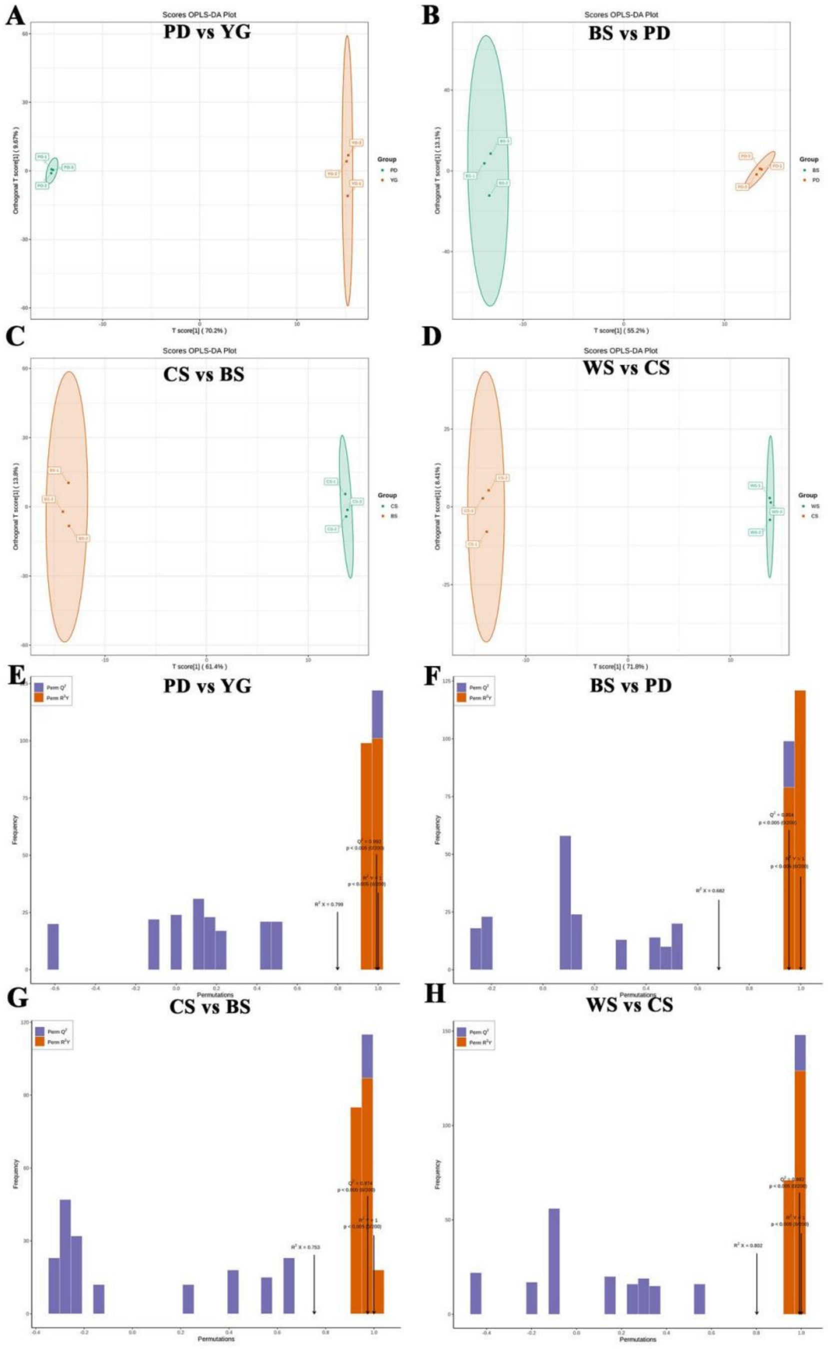

Figure S3. (A-D) Orthogonal partial least squares discriminant analysis (OPLS-DA) scores plots showing differentially accumulated metabolites (DAMs) in pairwise comparisons: (A) PD vs. YG, (B) BS vs. PD, (C) CS vs. BS, (D) WS vs. CS. (E-H) OPLS-DA model permutation in pairwise comparisons: (E) PD vs. YG, (F) BS vs. PD, (G) CS vs. BS, (H) WS vs. CS.

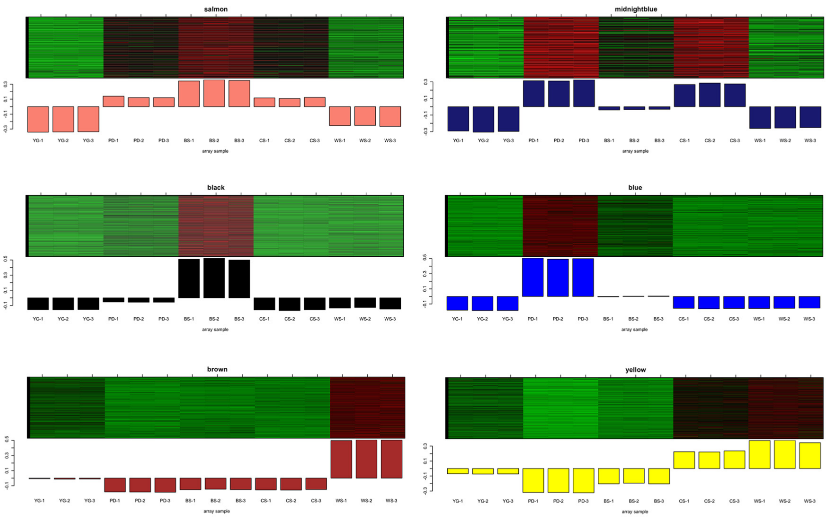

Figure S4. Gene expression patterns of salmon, midnightblue, black, blue, brown, and yellow modules by WGCNA, respectively.

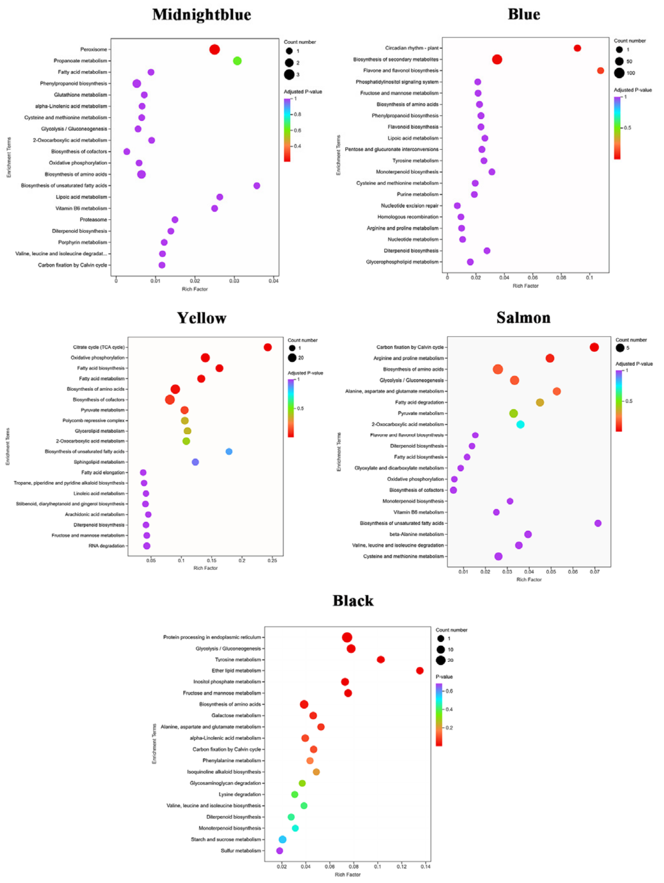

Figure S5. KEGG analysis of midnightblue, blue, yellow, salmon, and black modules by WGCNA, respectively. The size of dots represents the number of metabolites enriched in each pathway, and the color indicates the significance level.
